# Adverse Events of Single Balloon Enteroscopy‐assisted Endoscopic Retrograde Cholangiopancreatography in the Elderly: A Propensity Score Matching Analysis

**DOI:** 10.1002/deo2.70193

**Published:** 2025-08-26

**Authors:** Hiroki Nakagawa, Tsuyoshi Takeda, Takeshi Okamoto, Takafumi Mie, Takaaki Furukawa, Takashi Sasaki, Masato Ozaka, Takahisa Matsuda, Yoshinori Igarashi, Naoki Sasahira

**Affiliations:** ^1^ Department of Hepato‐Biliary‐Pancreatic Medicine Cancer Institute Hospital of Japanese Foundation for Cancer Research Tokyo Japan; ^2^ Department of Internal Medicine, Division of Gastroenterology and Hepatology, Omori Medical Center Toho University Tokyo Japan

**Keywords:** aged, cholangiopancreatography, complications, conscious sedation, single‐balloon enteroscopy

## Abstract

**Background:**

Sedation for conventional endoscopic retrograde cholangiopancreatography (ERCP) has been reported to be safe even for elderly patients. However, the safety of sedation for balloon enteroscopy‐assisted ERCP (BE‐ERCP) has not been well‐studied in the elderly.

**Methods:**

We retrospectively analyzed consecutive patients with surgically altered anatomy who underwent their initial BE‐ERCP using midazolam and pethidine at our institution between January 2016 and December 2022. The primary outcome was the rate of cardiopulmonary complications, including hypotension, bradycardia, tachycardia, and hypoxemia. Secondary outcomes included the rates of procedural interruptions, delayed arousal, postprocedural falls, and delirium, dose of sedatives, and rate of ERCP‐related adverse events (AEs). Risk factors for cardiopulmonary complications and ERCP‐related AEs were also examined.

**Results:**

A total of 190 patients were included (elderly group: 63; non‐elderly group: 127). After propensity score matching, 55 patients were selected for each group. The total dose of midazolam was significantly lower in the elderly group (4 mg vs. 5 mg, *p* = 0.033). Rates of cardiopulmonary complication (11% vs. 11%, *p* > 0.999) and ERCP‐related AEs (6% vs. 9%, *p* = 0.716) were not significantly different between the two groups. The total dose of pethidine (> 35 mg) and total procedure time (≥ 51 min) were identified as risk factors for cardiopulmonary complications and ERCP‐related AEs, respectively.

**Conclusions:**

Cardiopulmonary complications and ERCP‐related AEs were similar in elderly and non‐elderly patients undergoing BE‐ERCP. With careful monitoring, sedation during BE‐ERCP appeared safe, even for elderly patients.

## 1 Introduction

In Japan, the population aged ≥ 75 years is expected to account for 26.9% in 2060 due to the aging and declining birth rates [1]. Pancreaticobiliary diseases, including cholelithiasis [2], biliary tract cancer [3, 4], and pancreatic cancer [5], are therefore expected to increase. There is a growing demand for endoscopic retrograde cholangiopancreatography (ERCP) procedures in elderly patients. Sedation is useful to reduce patient discomfort during ERCP [6]. However, comorbidities and impaired organ function make elderly patients more sensitive to sedatives, creating a higher risk of sedation‐related adverse events (AEs) compared to non‐elderly patients [7, 8, 9]. Thus, the American Society for Gastrointestinal Endoscopy guidelines recommend the administration of fewer sedatives at a slower rate and with lower initial and cumulative doses for the elderly population [10].

Although several retrospective studies have shown that sedation for conventional ERCP can be safely performed in elderly patients [11, 12, 13], others have reported increased rates of hypoxemia [14], hypotension, or delayed arousal [15] in elderly patients. Recently, balloon enteroscopy‐assisted ERCP (BE‐ERCP) has been recognized as a standard treatment modality for pancreaticobiliary diseases in patients with surgically altered anatomy [16]. As BE‐ERCP is more complex and time‐consuming than conventional ERCP, the risk of sedation‐related AEs, including cardiopulmonary complications, may be higher in elderly patients undergoing BE‐ERCP. However, no studies to date have examined the safety of sedation in this population.

Therefore, we conducted this retrospective study to compare the safety of BE‐ERCP between elderly patients and non‐elderly patients, with a focus on sedation‐related AEs.

## 2 Methods

### 2.1 Patients

Consecutive patients with surgically altered anatomy who underwent BE‐ERCP at our institution between January 2016 and December 2022 were identified from our prospectively maintained database. Patients who received midazolam and pethidine for sedation were included in this study. Only the first BE‐ERCP procedure during the study period was evaluated for each patient. Patients who (1) required the introduction of new cardiovascular agents after admission and before ERCP, (2) received dexmedetomidine, or (3) underwent BE‐ERCP using a rendezvous technique were excluded. Patients were divided into two groups based on age: the elderly group (≥75 years) and the non‐elderly group (˂75 years). The cut‐off value for age was determined in accordance with the proposal from the Joint Committee of Japan Gerontological Society and the Japan Geriatrics Society [17]. This study was conducted in accordance with a master protocol (2023‐GB‐077) for gastrointestinal, hepatic, and pancreatobiliary diseases, which was approved by the institutional review board of our institution. The study was publicized on the hospital website, and all patients were given the opportunity to opt out.

### 2.2 Endoscopic Procedures

BE‐ERCP was performed using a short‐type single‐balloon enteroscope (SIF‐Y0015, or SIF‐H290S; Olympus, Tokyo, Japan) under conscious sedation with intravenous pethidine and midazolam. Carbon dioxide was used for insufflation during BE‐ERCP. After reaching the target site, biliary or pancreatic cannulation was generally attempted using a tapered catheter (MTW ERCP catheter; MTW Endoskopie, Wesel, Germany) or a sphincterotome (TRUEtome; Boston Scientific, Massachusetts, MA) and a 0.025‐inch guidewire (Visiglide2; Olympus, Tokyo, Japan or Endoselector; Boston Scientific, Massachusetts, MA). Endoscopic sphincterotomy or endoscopic balloon dilation was generally performed for patients with naïve papillas, while endoscopic balloon dilation was generally performed for those with biliary or pancreatic strictures. Basket or balloon catheters were used for stone removal, and plastic or metal stents were placed for biliary or pancreatic strictures as needed. All BE‐ERCP procedures were performed by expert endoscopists with more than five years of experience in therapeutic endoscopy or trainees under their direct supervision.

### 2.3 Details on Sedation and Monitoring

Moderate sedation was performed by the endoscopists using a combination of intravenous pethidine and midazolam [18]. The initial dose of pethidine was 35 mg in all patients, while that of midazolam was generally 3 mg, but was adjusted according to age and body size. Additional doses of either agent were injected at the discretion of the endoscopist.

All patients received intranasal oxygen (3 L/min), and their vital signs were checked before starting sedation. After starting sedation, blood pressure, heart rate, electrocardiogram, and percutaneous oxygen saturation were continuously monitored and recorded every 2–5 min.

### 2.4 Outcome Measurements and Definitions

The primary outcome was the rate of cardiopulmonary complications. Evaluated cardiopulmonary complications included hypotension (defined as systolic blood pressure <90 mmHg), bradycardia (defined as heart rate <50 beats/min), tachycardia (defined as heart rate > 120 beats/min), and hypoxemia (defined as SpO_2_ <90% under intranasal oxygen (3 L/min). For patients with systolic blood pressure <100 mmHg, heart rate >100 beats/min, or heart rate <60 beats/min before starting sedation, cardiopulmonary complications were defined as a change of 20% or more from baseline. Secondary outcomes included the rates of procedural interruptions due to cardiopulmonary complications, delayed arousal, postprocedural falls, and delirium; dose of sedatives, and rate of ERCP‐related AEs. Delayed arousal was defined as persistent somnolence 3 h after the conclusion of ERCP. Postprocedural falls and delirium were counted as events when occurring within 24 h after the conclusion of ERCP. The severity of ERCP‐related AEs was graded according to the American Society of Gastrointestinal Endoscopy lexicon guidelines [19] and the AEs in gastrointestinal endoscopy (AGREE) classification [20]. Age‐adjusted Charlson comorbidity index [21] and American Society of Anesthesiologists physical status [22] were used to evaluate the patient's general condition. Respiratory disease was defined as chronic obstructive pulmonary disease, sleep apnea syndrome, or bronchial asthma. Follow‐up data were collected up to 30 days after the index procedure.

### 2.5 Statistical Analysis

Continuous variables were expressed as median (range) and compared using the Mann‐Whitney U test. Categorical variables were expressed as absolute numbers (proportions) and analyzed using either the χ2 test or Fisher's exact test as appropriate. One‐to‐one propensity score matching without replacement was performed with a 0.2 caliper width to reduce the influence of possible confounding factors. A propensity score was estimated using a logistic regression model with the following factors: type of surgery, presence of cholangitis, and indications for BE‐ERCP. The balance between the two groups before and after matching was assessed using standardized mean differences. Logistic regression analysis was performed in the entire cohort to evaluate risk factors for cardiopulmonary complications and ERCP‐related AEs. Factors with *p*‐values < 0.10 were included in the multivariate analysis. *p*‐Values < 0.05 were considered statistically significant. Statistical analyses were performed using EZR software version 1.68 [23].

## 3 Results

### 3.1 Patient Characteristics

**FIGURE 1:**
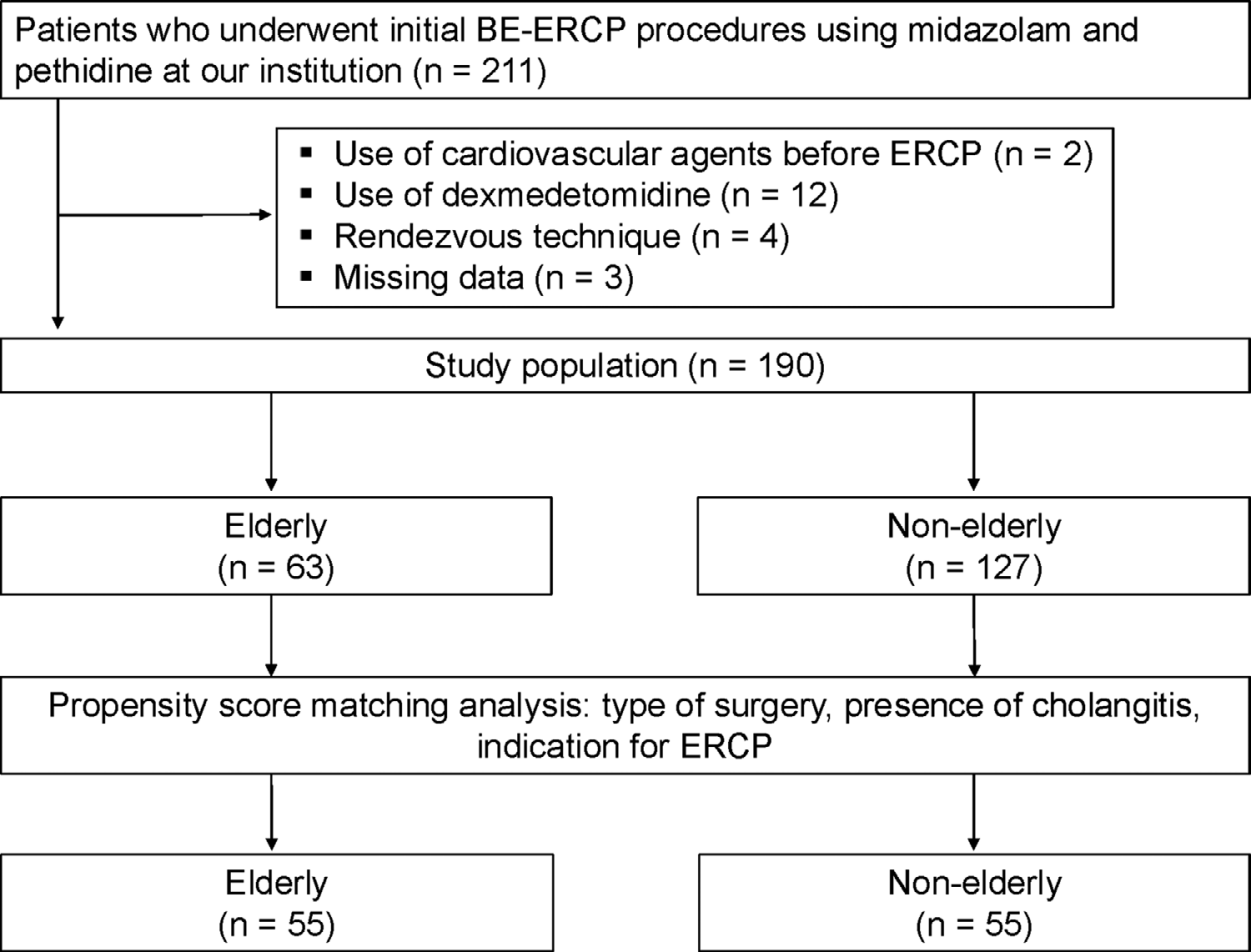
Patient flowchart. BE, balloon enteroscopy‐assisted; ERCP, endoscopic retrograde cholangiopancreatography.

A total of 211 patients who underwent their initial BE‐ERCP at our institution were identified from our database. Twenty‐one patients were excluded, and the remaining 190 patients were included in our analysis (Figure 1). Of the 190 patients, 63 were aged 75 years or older (elderly group) and 127 were less than 75 years old (non‐elderly group). Patient characteristics of each group are shown in Table 1. In the entire cohort, significant differences were observed in age, body weight, age‐adjusted Charlson comorbidity index, type of surgery, indication for BE‐ERCP, creatinine clearance, and systolic blood pressure. After propensity score matching, 55 patients were selected from each group, and the three covariates were well balanced. In the propensity score matching cohort, significant differences remained with respect to age (78 vs. 66 years, *p* < 0.001), body weight (53 vs. 58 kg, *p* = 0.033), age‐adjusted Charlson comorbidity index (7 vs. 5, *p* < 0.001), and creatinine clearance (57.5 vs. 74.2 mL/min, *p* < 0.001).

**TABLE 1:** Baseline characteristics of elderly and non‐elderly patients.

	Entire cohort *n =* 190				Propensity score matching cohort *n =* 110			
	Elderly *n =* 63	Non‐elderly *n =* 127	*p*‐Value	SMD	Elderly *n =* 55	Non‐elderly *n =* 55	*p*‐Value	SMD
Age, years	78 (75–89)	66 (38–74)	<0.001	2.384	78 (75–89)	66 (38–74)	<0.001	2.436
Sex, male	45 (71%)	90 (71%)	>0.999	0.012	40 (73%)	47 (86%)	0.159	0.317
Body weight, kg	53 (33–77)	58 (36–95)	0.023	0.379	53 (33–77)	58 (36–95)	0.033	0.443
ECOG performance status, 0/1	49 (78%)/ 11 (18%)	107 (84%)/ 20 (16%)	0.078	0.324	43 (78%)/ 10 (18%)	47 (86%)/ 8 (15%)	0.525	0.299
Age‐adjusted Charlson comorbidity index	7 (4–12)	5 (1–12)	<0.001	0.742	7 (4–12)	5 (2–10)	<0.001	0.777
ASA‐PS, I/II/III/IV	2/51/10/0	8/102/17/0	0.687	0.158	1/44/10/0	1/46/8/0	0.898	0.099
Comorbidities								
Hypertension	37 (59%)	58 (46%)	0.123	0.264	33 (60%)	23 (42%)	0.086	0.370
Diabetes mellitus	22 (35%)	36 (28%)	0.404	0.142	21 (38%)	20 (36%)	>0.999	0.038
Glaucoma	4 (6%)	10 (8%)	>0.999	0.059	4 (7%)	5 (9%)	>0.999	0.066
Heart disease	11 (18%)	16 (13%)	0.383	0.136	10 (18%)	10 (18%)	>0.999	<0.001
Chronic kidney disease	8 (13%)	6 (5%)	0.073	0.286	7 (13%)	4 (7%)	0.527	0.183
Respiratory disease	3 (5%)	15 (12%)	0.187	0.258	3 (6%)	6 (11%)	0.489	0.200
Liver cirrhosis	1 (2%)	0 (0%)	0.332	0.180	0 (0%)	0 (0%)	N/A	<0.001
Cerebrovascular disease	3 (5%)	3 (2%)	0.400	0.130	3 (6%)	2 (4%)	>0.999	0.087
Medications								
β‐blockers	5 (8%)	8 (6%)	0.762	0.064	4 (7%)	5 (9%)	>0.999	0.066
Anticoagulant drugs	10 (16%)	8 (6%)	0.062	0.309	8 (15%)	3 (6%)	0.202	0.307
Antiplatelet drugs	5 (8%)	9 (7%)	>0.999	0.032	3 (6%)	7 (13%)	0.320	0.255
Benzodiazepine	10 (16%)	17 (13%)	0.663	0.070	9 (16%)	7 (13%)	0.788	0.103
Type of surgery/reconstruction								
Gastrectomy/Roux‐en‐Y	32 (51%)	27 (21%)	<0.001	0.647	25 (46%)	24 (44%)	>0.999	0.037
Gastrectomy/Billroth‐II	0 (0%)	2 (2%)	>0.999	0.179	0 (0%)	0 (0%)	N/A	<0.001
Pancreaticoduodenectomy/Roux‐en‐Y	2 (3%)	9 (7%)	0.343	0.178	2 (4%)	2 (4%)	>0.999	<0.001
Pancreaticoduodenectomy/Billroth‐II	24 (38%)	73 (58%)	0.014	0.396	24 (44%)	25 (46%)	>0.999	0.037
Total pancreatectomy/Billroth‐II	1 (2%)	3 (2%)	>0.999	0.056	1 (2%)	2 (4%)	>0.999	0.112
Middle pancreatectomy/Roux‐en‐Y	0 (0%)	1 (1%)	>0.999	0.126	0 (0%)	0 (0%)	N/A	<0.001
Extrahepatic bile duct resection/Roux‐en‐Y	3 (5%)	12 (9%)	0.393	0.183	3 (6%)	2 (4%)	>0.999	0.087
Gastric bypass/Roux‐en‐Y	1 (2%)	0 (0%)	0.332	0.180	0 (0%)	0 (0%)	N/A	<0.001
Cholangitis	30 (48%)	61 (48%)	>0.999	0.008	29 (53%)	28 (51%)	>0.999	0.036
Indication for ERCP								
Suspicion of hepaticojejunostomy anastomotic stricture	24 (38%)	74 (58%)	0.013	0.412	24 (44%)	26 (47%)	0.848	0.073
Choledocholithiasis	32 (51%)	33 (26%)	0.001	0.528	28 (51%)	26 (47%)	0.849	0.073
Biliary stricture	4 (6%)	7 (6%)	>0.999	0.035	3 (6%)	2 (4%)	>0.999	0.087
Suspicion of pancreatic cancer	2 (3%)	1 (1%)	0.256	0.172	0 (0%)	1 (2%)	>0.999	0.192
Bile leak	1 (2%)	1 (1%)	>0.999	0.074	0 (0%)	0 (0%)	N/A	<0.001
Suspicion of pancreaticojejunostomy anastomotic stricture	0 (0%)	11 (9%)	0.017	0.435	0 (0%)	0 (0%)	N/A	<0.001
Blood findings								
Total bilirubin, mg/dL	0.9 (0.3–8.0)	0.9 (0.2–11.8)	0.551	0.051	0.9 (0.3–5.1)	1.2 (0.2–11.8)	0.340	0.250
AST, IU/L	59 (17–1265)	49 (13–1101)	0.379	0.056	65 (17–1265)	61 (14–1101)	0.837	0.250
Creatinine clearance, ml/min	57.0 (21.1–130.7)	76.3 (35.9–155.2)	<0.001	0.970	57.5 (21.1–130.7)	74.2 (41.3–155.2)	<0.001	0.880
CRP, mg/dL	1.6 (0.01–12.1)	1.0 (0.01–23.4)	0.677	0.017	1.9 (0.01–12.1)	1.5 (0.01–23.4)	0.327	0.082
Baseline vital signs								
Systolic blood pressure, mmHg	140 (98–201)	129 (84–193)	0.012	0.357	138 (98–201)	129 (84–193)	0.108	0.310
Heart rate, bpm	70 (47–116)	74 (48–129)	0.217	0.191	68 (47–116)	71 (48–107)	0.424	0.139
SpO_2_, %	98 (93–100)	98 (92–100)	0.923	0.002	98 (93–100)	98 (94–100)	0.400	0.060

Continuous variables are expressed as median (range), and categorical variables are expressed as absolute numbers (proportions).

Abbreviations: ASA‐PS, American Society of Anesthesiologists physical status; AST, aspartate aminotransferase; bpm, beats per minute; CRP, C‐reactive protein; ECOG, Eastern Cooperative Oncology Group; ERCP, endoscopic retrograde cholangiopancreatography; N/A, not available; SMD, standardized mean difference.

### 3.2 Outcome Measures

Details of procedural outcomes are shown in Table 2. Successful scope insertion (98% vs. 98%, *p* > 0.999) and total procedure time (50 vs. 53 min, *p* = 0.986) were comparable between the two groups. The initial dose (less than 3 mg; 40% vs. 5%, *p* < 0.001) and total dose (4 vs. 5 mg, *p* = 0.033) of midazolam were significantly lower in the elderly group, but there was no significant difference between the two groups in the total dose per kilogram of body weight. The initial dose and total dose of pethidine were comparable between the two groups. Flumazenil was administered to almost every patient at the end of the procedure (100% vs. 98%, *p* > 0.999). One patient in the elderly group received naloxone due to poor arousal.

**TABLE 2:** Endoscopic and sedation‐related outcomes.

	Elderly*n =* 55	Non‐elderly*n =* 55	*p*‐Value
Successful scope insertion	54 (98%)	54 (98%)	>0.999
Insertion time^a^, minutes	9 (3–65)	9 (3–58)	0.560
Total procedure time, minutes	50 (10–140)	53 (14–146)	0.986
Transpapillary approach	25 (46%)	24 (44%)	>0.999
Dose of midazolam			
Initial, mg/kg	0.05 (0.03–0.09)	0.05 (0–0.09)	0.045
Initial, mg			<0.001
<3	22 (40%)	3 (5%)	
≥ 3	33 (60%)	52 (95%)	
Total, mg/kg	0.08 (0.03–0.27)	0.09 (0.02–0.21)	0.162
Total, mg	4 (1–12)	5 (1–12)	0.033
Dose of pethidine			
Initial, 35mg	55 (100%)	55 (100%)	N/A
Total, mg			0.183
≤ 35	45 (82%)	38 (69%)	
> 35	10 (18%)	17 (31%)	
Scopolamine butylbromide	15 (27%)	18 (33%)	0.678
Flumazenil	55 (100%)	54 (98%)	>0.999
Naloxone hydrochloride	1 (2%)	0 (0%)	>0.999

Continuous variables are expressed as median (range) and categorical variables are expressed as absolute numbers (proportions).

N/A, not available.

^a^ Denominators adjusted to exclude patients with failed scope insertion.

Cardiopulmonary complications occurred in 24 patients in the entire cohort (elderly group: 8, non‐elderly group: 16). Outcomes of sedation‐related AEs in the propensity score matching cohort are shown in Table 3. The overall cardiopulmonary complication rates were not significantly different between the two groups (11% vs. 11%, *p* > 0.999). Hypotension occurred in 6% of patients in both the elderly and non‐elderly groups (*p* > 0.999). All patients were treated with fluid resuscitation, and none required vasopressors. Bradycardia occurred in 6% of patients in the elderly group, but did not occur in the non‐elderly group (p = 0.243). Atropine sulfate hydrate was given to one patient in the elderly group. There were no significant differences between the two groups in the rates of tachycardia (0% vs. 4%, *p* = 0.495) and hypoxemia (0% vs. 2%, *p* > 0.999). One patient in the elderly group experienced delayed arousal. Procedural interruptions, postprocedural falls, and delirium were not observed in either group.

**TABLE 3:** Sedation‐related adverse events.

	Elderly *n =* 55	Non‐elderly *n =* 55	*p*‐Value
**Cardiopulmonary complications**	6 (11%)	6 (11%)	>0.999
Hypotension	3 (6%)	3 (6%)	>0.999
Use of catecholamines	0	0	
Bradycardia	3 (6%)	0 (0%)	0.243
Use of atropine sulfate hydrate	1	0	
Tachycardia	0 (0%)	2 (4%)	0.495
Hypoxemia	0 (0%)	1 (2%)	>0.999
**Procedure terminated due to the above**	0 (0%)	0 (0%)	N/A
**Delayed arousal**	1 (2%)	0 (0%)	>0.999
**Postprocedural falls**	0 (0%)	0 (0%)	N/A
**Delirium**	0 (0%)	0 (0%)	N/A

Categorical variables are expressed as absolute numbers (proportions).

Abbreviation: N/A, not available.

ERCP‐related AEs occurred in 18 patients in the entire cohort (elderly group: four, non‐elderly group: 14). Outcomes of ERCP‐related AEs in the propensity score matching cohort are shown in Table 4. The overall ERCP‐related AE rates were not significantly different between the two groups (6% vs. 9%, *p* = 0.716). Two patients in the elderly group developed moderate post‐ERCP pancreatitis, which improved with conservative treatment. Two patients in the non‐elderly group developed perforation of the bile duct. One case occurred following endoscopic sphincterotomy and endoscopic papillary large balloon dilation, while the other occurred following balloon dilation for hepaticojejunostomy anastomotic stricture. Both patients were successfully managed with conservative treatment. All ERCP‐related AEs were classified as Grade II according to the AGREE classification.

**TABLE 4:** Endoscopic retrograde cholangiopancreatography (ERCP)‐related adverse events.

	Elderly *n =* 55	Non‐elderly *n =* 55	*p*‐Value
**ERCP‐related adverse events** ^a^	3 (6%)	5 (9%)	0.716
Perforation	0 (%)	2 (4%)	0.495
Mild/moderate/severe		0/1/1	
Grade I/II/III/IV/V		0/2/0/0/0	
Cholangitis	1 (2%)	3 (6%)	0.618
Mild/moderate/severe	0/1/0	2/1/0	
Grade I/II/III/IV/V	0/1/0/0/0	0/3/0/0/0	
Cholecystitis	0 (0%)	0 (0%)	N/A
Pancreatitis	2 (4%)	0 (0%)	0.495
Mild/moderate/severe	0/2/0		
Grade I/II/III/IV/V	0/2/0/0/0		
Bleeding	0 (0%)	0 (0%)	N/A
Aspiration pneumonia	0 (0%)	0 (0%)	N/A

Categorical variables are expressed as absolute numbers (proportions).

Abbreviations: ERCP, endoscopic retrograde cholangiopancreatography; N/A, not available.

^a^ The severity of ERCP‐related adverse events was graded according to the American Society of Gastrointestinal Endoscopy lexicon guidelines (mild, moderate, or severe) and the adverse events in gastrointestinal endoscopy (AGREE) classification (Grades I–V).

Procedural outcomes and AEs stratified by the type of surgery for the entire cohort are shown in Tables S1 and S2. The scope insertion time and total procedure time were significantly longer in the Roux‐en‐Y group compared to the Billroth‐II group, which resulted in a higher total dose of midazolam and pethidine. Although hypotension was significantly more commonly observed in the Roux‐en‐Y group (10% vs. 2%, *p* = 0.025), ERCP‐related AE rates were similar between the two groups (10% vs. 9%, *p* = 0.805).

### 3.3 Risk Factors for Cardiopulmonary Complications and ERCP‐Related AEs

Univariate and multivariate logistic regression analyses of risk factors for cardiopulmonary complications and ERCP‐related AEs in the entire cohort are shown in Tables 5 and 6. Total pethidine dose greater than 35 mg (odds ratio [OR]: 2.63; 95% confidence interval [CI], 1.05–6.61; *p* = 0.039) and total procedure time greater than 51 min (OR: 8.20; 95% CI, 1.75–38.5; *p* = 0.008) were identified as risk factors for cardiopulmonary complications and ERCP‐related AEs, respectively.

**TABLE 5:** Logistic regression analyses of risk factors for cardiopulmonary complications.

	Univariate			Multivariate		
	Odds ratio	95% CI	*p*‐Value	Odds ratio	95% CI	*p*‐Value
Cardiopulmonary complications						
Age						
≥75 years	1.01	0.41–2.50	0.984			
Body weight						
≥56 kg	1.15	0.49–2.72	0.744			
Age‐adjusted Charlson comorbidity index						
≥6	0.56	0.24–1.32	0.185			
ASA‐PS						
≥3	0.85	0.23–3.05	0.798			
Hypertension						
Yes	1.21	0.51–2.86	0.663			
Heart disease						
Yes	0.85	0.23–3.05	0.798			
Respiratory disease						
Yes	1.44	0.38–5.39	0.590			
Cerebrovascular disease						
Yes	3.68	0.64–21.30	0.145			
Use of benzodiazepine						
Yes	1.24	0.39–3.97	0.713			
Use of β‐blockers						
Yes	1.28	0.27–6.17	0.757			
Type of surgery						
Roux‐en‐Y	2.68	1.09–6.60	0.033	2.09	0.80–5.45	0.133
Cholangitis						
Yes	0.91	0.39–2.15	0.829			
Creatinine clearance						
≤72 mL/min	0.83	0.35–1.95	0.663			
Total midazolam						
≥5 mg	1.69	0.69–4.16	0.255			
Total pethidine						
>35 mg	3.26	1.35–7.83	0.008	2.63	1.05–6.61	0.039
Total procedure time						
≥51 min	2.20	0.89–5.43	0.086	1.36	0.51–3.66	0.538

Abbreviations: ASA‐PS, American Society of Anesthesiologists physical status; CI, confidence interval.

**TABLE 6:** Logistic regression analyses of risk factors for endoscopic retrograde cholangiopancreatography (ERCP)‐related adverse events in the entire cohort.

	Univariate			Multivariate		
	Odds ratio	95% CI	*p*‐Value	Odds ratio	95% CI	*p*‐Value
ERCP‐related adverse events						
Age						
≥ 75 years	0.55	0.17–1.74	0.306			
Age‐adjusted Charlson comorbidity index						
≥ 6	0.88	0.33–2.34	0.796			
Type of surgery						
Roux‐en‐Y	1.21	0.46–3.18	0.707			
Indication for ERCP						
Choledocholithiasis	2.66	1.00–7.11	0.051	1.48	0.52–4.18	0.459
Transpapillary approach						
Yes	1.04	0.37–2.90	0.947			
Total procedure time						
≥ 51 min	9.42	2.10–42.2	0.003	8.20	1.75–38.5	0.008

Abbreviations: CI, confidence interval; ERCP, endoscopic retrograde cholangiopancreatography.

## 4 Discussion

In this study, we evaluated the safety of sedation during BE‐ERCP in elderly patients (≥75 years) compared to non‐elderly patients (˂75 years) using propensity score matching analysis. Total dose of midazolam was significantly lower in the elderly group (4 mg vs. 5 mg, *p* = 0.033). Rates of cardiopulmonary complications (11% vs. 11%, *p* > 0.999) and ERCP‐related AEs (6% vs. 9%, *p* = 0.716) were not significantly different between the two groups. Total dose of pethidine (>35 mg) and total procedure time (≥51 min) were identified as risk factors for cardiopulmonary complications and ERCP‐related AEs, respectively.

Several studies have reported on the safety of sedation for conventional ERCP in elderly patients [11, 12, 13, 14, 15, 24], albeit with inconsistent results. This may be attributable to differences in the definition of elderly patients and cardiopulmonary complications, procedure time, and the type of sedatives used in each study. Yang et al. [11] conducted a retrospective study on 792 patients, comparing elderly patients (≥80 years) to non‐elderly patients (<65 years) who underwent ERCP with midazolam and propofol. Cardiopulmonary complications in the study were defined as follows: hypotension, systolic blood pressure <90 mmHg; bradycardia, heart rate <50 beats/min; tachycardia, heart rate >120 beats/min; hypoxemia, SpO_2_< 90% under intranasal oxygen (2 L/min). While the mean dose of propofol used was lower in the elderly group, rates of cardiopulmonary complications were similar between the two groups (2.2% vs. 1.0%, *p* = 0.16). Finkelmeier et al. [24] retrospectively reviewed 758 patients who underwent ERCP with propofol, midazolam, or ketamine. Although the dose of sedatives used was lower in the elderly (>80 years), rates of cardiopulmonary complications were significantly higher in the elderly than in the non‐elderly (≤ 80 years; 3.4% vs. 0.5%, *p* < 0.05).

In general, BE‐ERCP is considered more complex and time‐consuming than conventional ERCP and often requires deeper sedation. Therefore, the findings of prior studies on conventional ERCP may not be directly applicable to BE‐ERCP. In this study, the rates of cardiopulmonary complications were higher than those reported in previous studies on conventional ERCP [11, 12]. Roux‐en‐Y reconstruction is considered one of the factors that contribute to procedural failure in BE‐ERCP because it is difficult to reach the target site [25]. Although the total amount of pethidine and midazolam and rates of cardiopulmonary complications were significantly higher in patients with Roux‐en‐Y reconstruction compared to those with Billroth‐II reconstruction, rates of cardiopulmonary complications were not affected by the type of reconstruction in the multivariate analysis. Furthermore, rates of cardiopulmonary complications were not significantly different between elderly and non‐elderly patients (11% vs. 11%, *p* > 0.999) in this study. This may be explained by the lower amount of midazolam used in the elderly group. Although sedation during BE‐ERCP in elderly patients appeared safe, delayed arousal after the procedure may require attention. One elderly patient aged 87 years who required 7 mg of midazolam and 35 mg of pethidine for a 120‐min procedure experienced persistent somnolence 3 h after the conclusion of ERCP. As the effects of midazolam depend on the dose and duration of its administration [26], reducing the amount of midazolam by adding other drugs, including opioids and dexmedetomidine, may lower the risk of sedation‐related AEs [27, 28].

Most studies on conventional ERCP have reported comparable rates of ERCP‐related AEs between elderly and non‐elderly patients [11, 12, 15]. However, data on BE‐ERCP‐related AEs are limited. In this study, the incidence of ERCP‐related AEs was not significantly different between groups (6% vs. 9%, *p* = 0.716), in line with previous reports on BE‐ERCP (5.8% to 12.0%) [16, 29, 30, 31]. Recently, Hakuta et al. [31] retrospectively reviewed 568 elderly (≥75 years) and non‐elderly patients (<75 years) who underwent BE‐ERCP. The incidence of early AEs (within 14 days after BE‐ERCP) was not significantly different between groups (12.0% vs. 9.0%, *p* = 0.31). The study showed that prolonged procedure time (>90 min) was a risk factor for early AEs (OR, 1.98; 95% CI, 1.08–3.60; *p* = 0.03), while age was not (OR, 1.36; 95% CI, 0.74–2.51; *p* = 0.32). In particular, aspiration pneumonia occurred in four elderly patients with long procedure times (median: 110 min). None of the patients in our study developed aspiration pneumonia, possibly due to the shorter procedure time. Thus, it may be necessary to consider terminating the procedure early to avoid this risk, particularly in the elderly.

There are several limitations in this study. First, this was a single‐center retrospective study with a limited sample size, and selection bias is inevitable. Although propensity score matching was used to reduce bias, unmeasured confounding factors may still exist. Second, there was no strict protocol for the administration of sedatives. The initial dose and intraprocedural addition of sedatives were left to the discretion of the treating endoscopist. Finally, the efficacy and safety of sedatives other than midazolam and pethidine, such as propofol or remimazolam, remain uncertain.

In conclusion, cardiopulmonary complications and ERCP‐related AEs were not affected by age in patients with surgically altered anatomy who underwent BE‐ERCP. With careful monitoring, sedation during BE‐ERCP appeared safe, even for elderly patients. Further studies with larger sample sizes are needed to confirm the results of this study.

## Ethics Statement

Approval of the research protocol by an Institutional Review Board: This study was conducted in accordance with a master protocol (2023‐GB‐077) for gastrointestinal, hepatic, and pancreatobiliary diseases, which was approved by the institutional review board of our institution.

## Consent

Informed consent for this study was waived due to its retrospective design. The study was publicized on the hospital website, and all patients were given the opportunity to opt out.

## Conflicts of Interest

Tsuyoshi Takeda has received a speaker honorarium from Boston Scientific Japan, Japan Lifeline, and SB‐Kawasumi Laboratories. Takashi Sasaki has received a speaker honorarium from Boston Scientific Japan, Century Medical, Kaneka Medix, Japan Lifeline, and SB‐Kawasumi Laboratories. Naoki Sasahira has received consulting fees from Gadelius Medical and a speaker honorarium from Olympus Medical and Kaneka Medix.

## Clinical Trial Registration

N/A.

## Supporting information




**Supporting Table 1**: Endoscopic and sedation‐related outcomes by type of surgery.

**Supporting Table 2**: Sedation‐related adverse events and ERCP‐related adverse events by type of surgery.
