# Supplementary material for: Adverse Events of Single Balloon Enteroscopy‐assisted Endoscopic Retrograde Cholangiopancreatography in the Elderly: A Propensity Score Matching Analysis
Source: DEN Open. 2025 Aug 26;6(1):e70193. doi: 10.1002/deo2.70193 (PMC12378559; doi:10.1002/deo2.70193)
Supplement: Supplementary file 1 — Supporting Table 1: Endoscopic and sedation‐related outcomes by type of surgery. [file DEO2-6-e70193-s001.docx]

**Supplementary Table 1.** Endoscopic and sedation-related outcomes by type of surgery.

|  | **Roux-en-Y**  **n = 87** | **Billroth-II**  **n = 103** | **P value** |
| --- | --- | --- | --- |
| Successful scope insertion | 82 (94%) | 101 (98%) | 0.250 |
| Insertion time^a^, minutes | 12 (3–89) | 7 (2–58) | < 0.001 |
| Total procedure time, minutes | 60 (20–146) | 40 (9–87) | < 0.001 |
| Dose of midazolam  ▪︎ Initial, mg/kg  ▪︎ Initial, mg  < 3  ≥ 3  ▪︎ Total, mg/kg  ▪︎ Total, mg | 0.05 (0.03–0.09)  18 (21%)  69 (79%)  0.10 (0.03–0.27)  5.0 (2.0–12.0) | 0.05 (0–0.09)  17 (17%)  86 (83%)  0.08 (0.02–0.18)  4.5 (1.0–10.0) | 0.989  0.574  0.003  0.005 |
| Dose of pethidine  ▪︎ Initial, 35mg  ▪︎ Total, mg  ≤ 35  > 35 | 87 (100%)  56 (64%)  31 (36%) | 103 (100%)  83 (81%)  20 (19%) | N/A  0.014 |

Continuous variables are expressed as median (range) and categorical variables are expressed as absolute numbers (proportions).

N/A, not available.

^a^ Denominators adjusted to exclude patients with failed scope insertion.
