# Supplementary material for: Adverse Events of Single Balloon Enteroscopy‐assisted Endoscopic Retrograde Cholangiopancreatography in the Elderly: A Propensity Score Matching Analysis
Source: DEN Open. 2025 Aug 26;6(1):e70193. doi: 10.1002/deo2.70193 (PMC12378559; doi:10.1002/deo2.70193)
Supplement: Supplementary file 2 — Supporting Table 2: Sedation‐related adverse events and ERCP‐related adverse events by type of surgery. [file DEO2-6-e70193-s002.docx]

**Supplementary Table 2.** Sedation-related adverse events and ERCP-related adverse events by type of surgery.

|  | **Roux-en-Y**  **n = 87** | **Billroth-II**  **n = 103** | **P value** |
| --- | --- | --- | --- |
| Cardiopulmonary complications^a^ | 16 (18%) | 8 (8%) | 0.047 |
| ▪ Hypotension | 9 (10%) | 2 (2%) | 0.025 |
| ▪︎ Bradycardia | 4 (5%) | 4 (4%) | > 0.999 |
| ▪︎ Tachycardia | 4 (5%) | 2 (2%) | 0.415 |
| ▪︎ Hypoxemia | 1 (1%) | 1 (1%) | > 0.999 |
| Procedure terminated due to the above | 2 (2%) | 0 (0%) | 0.208 |
| Delayed arousal | 1 (1%) | 0 (0%) | 0.458 |
| Postprocedural falls | 0 (0%) | 0 (0%) | N/A |
| Delirium | 0 (0%) | 0 (0%) | N/A |
| ERCP-related adverse events^b^ | 9 (10%) | 9 (9%) | 0.805 |
| ▪︎ Perforation  Mild/moderate/severe  Grade Ⅰ/Ⅱ/Ⅲ/Ⅳ/V | 1 (1%)  0/0/1  0/1/0/0/0 | 1 (1%)  0/1/0  0/1/0/0/0 | > 0.999 |
| ▪︎ Cholangitis^c^  Mild/moderate/severe  Grade Ⅰ/Ⅱ/Ⅲ/Ⅳ/V | 6 (7%)  5/1/0  0/6/0/0/0 | 7 (7%)  6/1/0  0/6/1/0/0 | > 0.999 |
| ▪︎ Cholecystitis | 0 (0%) | 0 (0%) | N/A |
| ▪︎ Pancreatitis  Mild/moderate/severe  Grade Ⅰ/Ⅱ/Ⅲ/Ⅳ/V | 2 (2%)  0/2/0  0/2/0/0/0 | 1 (1%)  0/1/0  0/1/0/0/0 | 0.594 |
| ▪︎ Bleeding | 0 (0%) | 0 (0%) | N/A |
| ▪︎ Aspiration pneumonia | 0 (0%) | 0 (0%) | N/A |

Categorical variables are expressed as absolute numbers (proportions).

N/A, not available; ERCP, endoscopic retrograde cholangiopancreatography.

^a^ One patient developed both hypotension and bradycardia, and one patient developed both bradycardia and hypoxemia in the Roux-en-Y group. One patient developed both hypotension and bradycardia in the Billroth-II group.

^b^ The severity of ERCP-related adverse events was graded according to the American Society of Gastrointestinal Endoscopy lexicon guidelines (mild, moderate, or severe) and the adverse events in gastrointestinal endoscopy (AGREE) classification (Grade Ⅰ-V).

^c^ One patient in the Billroth-II group underwent endoscopic nasobiliary drainage tube placement for cholangitis and was classified as grade IIIa according to the AGREE classification.
